# What shapes research impact on policy? Understanding research uptake in sexual and reproductive health policy processes in resource poor contexts

**DOI:** 10.1186/1478-4505-9-S1-S3

**Published:** 2011-06-16

**Authors:** Andy Sumner, Jo Crichton, Sally Theobald, Eliya Zulu, Justin Parkhurst

**Affiliations:** 1 Institute of Development Studies, University of Sussex, Brighton, UK; 2 African Population and Health Research Center (APHRC), Kenya; 3 Liverpool School of Tropical Medicine (LSTM), UK; 4 African Institute for Development Policy (AFIDEP), Kenya; 5 London School of Hygiene and Tropical Medicine (LSHTM), UK

## Abstract

Assessing the impact that research evidence has on policy is complex. It involves consideration of conceptual issues of what determines research impact and policy change. There are also a range of methodological issues relating to the question of attribution and the counter-factual. The dynamics of SRH, HIV and AIDS, like many policy arenas, are partly generic and partly issue- and context-specific. Against this background, this article reviews some of the main conceptualisations of research impact on policy, including generic determinants of research impact identified across a range of settings, as well as the specificities of SRH in particular. We find that there is scope for greater cross-fertilisation of concepts, models and experiences between public health researchers and political scientists working in international development and research impact evaluation. We identify aspects of the policy landscape and drivers of policy change commonly occurring across multiple sectors and studies to create a framework that researchers can use to examine the influences on research uptake in specific settings, in order to guide attempts to ensure uptake of their findings. This framework has the advantage that distinguishes between pre-existing factors influencing uptake and the ways in which researchers can actively influence the policy landscape and promote research uptake through their policy engagement actions and strategies. We apply this framework to examples from the case study papers in this supplement, with specific discussion about the dynamics of SRH policy processes in resource poor contexts. We conclude by highlighting the need for continued multi-sectoral work on understanding and measuring research uptake and for prospective approaches to receive greater attention from policy analysts.

## Introduction

This paper reviews existing literature to address the following two questions: What are the factors that typically influence the impact of public health research evidence on policy? And what are the specificities of research impact on policy in Sexual and Reproductive Health (SRH) policy in particular? Throughout this paper we use the term SRH to encompass HIV and AIDS, as well as other SRH issues such as sexuality, reproductive services and Sexually Transmitted Infection (STI) treatment.

During recent years, international public health researchers have been increasing their use of conceptual analysis, policy theory and policy analysis techniques in an effort to better understand health policy processes [1-4]. There have also been numerous calls for policy and decision makers to increase their uptake and use of research evidence and systematic reviews, with tools and models proposed to help guide these decision makers (see for instance the special issue of Implementation Science authored by Lavis and colleagues [5]). Much of this work is informed by concerns with ‘evidence-based policy’ more broadly in the field of social/public policy [6,7]. Finally, some efforts have been made in the international development community to develop models and frameworks that help explain the uptake of evidence into policy in low and middle income countries [6]. Of course, public advocacy groups may also at times call for greater use of evidence by policy makers, and indeed, governments’ stated commitment to the use of evidence may often follow from perceptions of public concerns.

From the bodies of literature engaging with these issues, there is enormous scope for greater cross-fertilisation of concepts in order to provide guidelines and recommendations to researchers in low and middle income countries. There is also a great deal of insight to be provided by the field of policy analysis – a sub field of political science dedicated to understanding the process of policy change and the factors influencing such change [8-10]. As most policy analysis work emphasises, context is particularly important to understanding policy change, and as such these guidelines must take into account the specificities of not only the local policy environment, but also the specifics of the research issue at hand.

Many researchers and communications specialists working on SRH have an interest in understanding the role of research evidence in SRH policy processes, but often lack knowledge of skills and approaches for analysing its impact [11]. While in many cases they are engaging with policy processes throughout their work and have considerable experiential knowledge of their specific situations, such individuals often have limited knowledge of the concepts, skills and frameworks needed for analysing their experiences and relating them to other case studies. This paper represents one such collaboration. Drawing together a team of authors from various disciplines and backgrounds, it combines approaches and insights from studies of research impact involving a range of sectors and settings in order to examine the challenge of improving and assessing the uptake of research in SRH policy.

It is not the intention of this article to provide a systematic review of the entire research impact on policy literature (that has been more comprehensively attempted by, among others, Boaz and colleagues [12]). Rather, we aim to introduce the papers in this special issue by providing an overview of the conceptual and empirical issues in the research impact literature with specific regard to public health, cognizant of the specific issues raised by SRH policy in particular. Further, we wish to provide professionals working in public health with an analytical framework for understanding the factors influencing research uptake, distinguishing between those factors related to the context where researchers are working and the actions that researchers themselves can take to increase impact. We apply this framework to the case study papers in this supplement and discuss the specificities of research impact on policy in the area of SRH.

It is clear that the impact of research evidence on policy and practice is an agenda that has been gathering momentum. This agenda represents a coming together of three divergent concerns. The first, from the funders of research, draws on results-based management and is concerned with getting value-for-money from research spending. The second, more typical of those in the development studies research community, is concerned with whether research in the area is ‘making a difference’. Among development researchers there is also often a normative basis – addressing global poverty and inequality, and catalysing change. The third, however, comes from policy makers who may often express frustration about, amongst other things: the seemingly over-theoretical nature of much research work; the tendency for researchers to simply appear out of nowhere with results in hand, hoping or assuming they will be taken up; and the often limited suggestions for implementation or operationalisation of issues of relevance.

It is also worth pointing out that ‘researchers’ are not the only group interested in research uptake and evidence-based policy making. Such guidelines and recommendations may also be of use to lobbyists, advocates and even potential policy beneficiaries. For example, at the Paulo Longo Research Initiative (PRLO), sex workers, alongside scholars and policy analysts, are demanding an improved evidence base and more attuned policy process in order to improve their health and wellbeing. The appeal of strengthened analytical work on research uptake and impact is therefore not limited to the formal researcher community.

In our paper, we focus specifically on evidence arising from organised research efforts (these can be theoretical, operational, or evaluative research efforts). Evidence can take many forms, and have multiple meanings, however. We recognise the debates around the contested nature of evidence, and the insights provided by authors such as Berridge and Stanton [13] who have shown the historical and social construction of ‘evidence based policy’, as well as authors such as Lin [14] who have illustrated that policy makers respond to multiple competing priorities to make decisions, not only considering evidence arising from scientific research. This understanding of the multitude of evidences from the perspective of the decision maker is a critical concept to include in policy analyses. However, the public health field has a much more established hierarchy of evidence types – with systematic reviews of randomised trials featuring at the top of the hierarchy, and anecdotal or individual cases towards the bottom. Policy makers may not share or use this categorisation of evidence – indeed a common trend in policy speeches is to invoke specific anecdotes or cases to sway an audience. But this paper is written for a public health community concerned with getting types of evidence deemed to be better quality, and methodologically robust, into policy and practice. We therefore accept a positivist bias in our approach to evidence here, primarily because of the audience we target. This does not mean we ignore the alternative views of evidence – indeed, an awareness of these alternative types of evidence is critical for understanding how research evidence is used – but it means when we speak of research evidence here, we refer to that which the public health community has agreed on as valid to act upon.

The dynamics of SRH – like many policy arenas – are partly generic and partly issue and context specific. As with other health-related issues, evidence typically seen to have policy relevance comes from well established fields of medicine, epidemiology, and public health. There are also often established structures or policy influencing institutions in place to which research findings must be targeted. In many cases, then, promoting the uptake of evidence into policy may mean identifying the correct institutions, structures, networks and individuals with whom to link in order to ensure consideration. However, policy issues relating to SRH can also be highly politicised and sensitive, requiring a range of additional approaches or partnerships more explicitly addressing the political nature of decision making in order to ensure research engagement. Historically there have been shifts in global prioritising of different SRH issues, and the priority placed on SRH issues on the policy agenda may vary across settings. In many contexts, past concerns with reproductive health have fallen in attention, with many sexual and reproductive health issues conceptualised by policy makers and practitioners as low priority and low profile. Meanwhile, HIV and AIDS, originally highly stigmatised and avoided by many policy makers, has risen up national and international policy agendas in recent years to attract unprecedented levels of financial support. Furthermore, priorities may vary not just between broad SRH categories as just mentioned, but also *within* them. For example, in the Middle East and North Africa (MENA) region there are a number of areas of HIV work that remain highly politically unpalatable, such as the vulnerability of men who have sex with men. These levels of political prioritisation at particular points in time set the context in which decision making on SRH issues is made. It is therefore necessary to recognise that across the whole health sector, the political economy of health financing and management cannot be ignored and may present challenges to the use of research in policy.

The paper is structured as follows. Section 2 provides an overview of the conceptual issues in research impact and policy change with a focus on the health sector. Section 3 introduces an analytical framework for factors shaping research impact on policy (presented in Table 1). Section 4 focuses on the specificities of SRH and applies this analytical framework to the case study papers in this supplement (see Additional file 1). Section 5 concludes.

**Table 1:** An analytical framework for factors shaping research impact on policy

What determines policy outcomes?	Factors shaping research impact on policy	
	
	‘Pre-conditions’ affecting research impact on policy	Actions and strategies to increase probability of research impact on policy
Policy ideas, narratives and discourse(s)	Extent to which there is a consensus on the nature of the problem and appropriate responses	Packaging of research or ‘knowledge translation’ for policy audience – e.g. explicit and clear policy recommendations; short summaries or briefs; using policy ‘language’ such as economic vocabulary, framing of research to resonate with prevailing policy discourses, or tailoring messages to specific policy environments.
	
	Extent of influence of international discourses on domestic policy	Research methodologies that develop research user ‘ownership’ throughout the research process.
	
	Extent to which policy issue is novel	Explicit, targeted communication and dissemination strategies.

Policy actors and networks	Extent to which ruling party is ideologically driven	Interpersonal relationships and networks - Building or connecting to policy networks; policy ‘champions’ and intermediaries and consultations with key policy actors on research during project.
	
	Extent of ‘special interests’ or range of actors - such as service users, the private sector, unions, or professional associations; or strength of civil society, or influence of donors in policy arena.	Credibility or ‘brand’ of the originating institution, funder or researcher(s).
	
	Level of bureaucracy, professionalism and capacity to process evidence.	Extent of ‘border-crossing’ between research and policy communities
	
	Importance placed on systematic and other evidence reviews by policy makers in power *	Utilising knowledge brokers to specifically get research to policy makers

Context and institutions	Extent of democratic openness; degree of academic and media freedom; norms on consultation and participation in policy processes.	Planning research to align to specific timing of expected ‘policy windows’ – e.g. research aimed at important meetings of officials/politicians.
	
	Use of multi-year development plans and other planning instruments	Planning research to align to ready existing or created ‘policy spaces’ – electoral spaces; consultative spaces; popular protest spaces, etc.
	
	Level of centralisation of political decision making	Framing of research around unexpected events – e.g. the financial crisis; need for public expenditure efficiency, etc.
	
	Established institutional structures and policy advisory bodies which exist to link researchers and policy makers*	Working creatively with these structures throughout the research cycle

Source: Adapted from [16,45,48].

* See [5,49,50].

### The conceptualisation of research impact and policy change

Studying the impact of research on policy requires conceptualising outcome measures – including the nature of research ‘impact’ and policy change – as well as understanding the determinants of policy change itself. In terms of the research impact on policy, Davies and colleagues explain:

Non-academic research impact is about identifying the influences of research findings on policy, managerial and professional practices, social behaviour or public discourse. Such impact may be instrumental, influencing changes in policy, practices and behaviour, or conceptual, changing people’s knowledge, understanding and attitudes towards social issues…research can contribute not just to decisional choices, but also to the formation of values, the creation of new understandings and possibilities, and to the quality of public and professional discourse and debate [15].

Instrumental and conceptual uses may be intricately entwined in policy processes. An example from SRH might be how changing attitudes to specific sub-populations, such as sex workers or young people, could affect how health services are designed and targeted, which in turn may affect the behaviour and practice of health workers.

Jones and Sumner [16] explore the different ways research may have an impact on various aspects of policy and policy processes. These are through:

▪ *Agenda setting* – changes in policy makers’ priorities and attention to new or previously under-emphasized policy issues (for example, following research showing the growing size of urban versus rural population, growing urban poverty and lack of reproductive health services among the urban poor, the Gates Foundation has made a sizable investment in implementation research to demonstrate ways of improving such services), and *shifts in policy framing* – changes in the way that policy makers understand a problem or the possible responses to it (for example, efforts by NGOs to ‘reframe’ reproductive rights to increase their acceptance and legitimacy among policy makers [17]).

▪ *Changes in the content of policy* – substantive change in the content of policy and/or resources allocated (for example, the introduction of ARVs in HIV treatment guidelines in developing countries) [18,19].

▪ *Changes in the way policy is delivered* – substantive change in the way policy is implemented and/or the way policy is delivered to intended recipients (for example, research on the need for greater donor coordination or increased voice for service users may influence the ways in which decisions are made in the health sector). Sometimes new health evidence can impact on practice first and later become integrated into policy, as with the adoption of new drugs to treat STIs by doctors before a change in national STI treatment policy [20,21].

In practice these policy impacts may overlap too. For example, research which leads to the abolition of a particular ‘failing’ programme could be conceptualised as both a change in policy content and in policy implementation.

With an understanding of the kinds of impacts research may have on policy, it is then necessary to examine the factors that influence policy change. Research relating to decision-making in public policy processes in general has evolved from Northern contexts since Lasswell and Lerner [22], and particularly so in the 1970s/1980s [10,23-27]. Such research has been expanded to Southern contexts over the last two decades [1,28-35].

The net result is that there is now a bewildering array of theories and analytical frameworks that attempt to describe policy processes or explain factors influencing policy change (of which research will be only one element). In terms of health policy analysis specifically, there is a rich literature on policy processes which is broadly consistent with policy studies from other fields. Walt and Gilson’s classic work on the subject emphasises the importance of considering context, processes, and the central role of actors in explaining policy content change [1]. More recent works have built on this to go further into the importance of actor networks, power and interests, and multiple conceptualisations of the policy process – from a simple staged model to one drawing on Paul Sabatier’s and Christopher Weible’s ‘advocacy coalitions framework’ which sees the process of policy change reflecting an ongoing struggle between opposed groups [2,36-39].

The field of health policy studies has also attempted to grapple specifically with issues of the use of evidence in policy. Innvaer and colleagues conducted a review of studies on health policy-makers’ views of evidence-usage, identifying 24 studies which had conducted interviews with health policy makers [40]. 21 of the 24 studies examined the role of evidence in actual decision-making processes, whilst the remaining 3 examined hypothetical questions. Only 4 of the 24 studies were conducted in low and middle-income countries (Pakistan, Burkina Faso, South Africa and Mexico). Both facilitators and barriers identified across the developing country studies related not just to the supply of, but also the demand for, evidence. This, again, emphasises the importance of understanding policy processes in context, and not assuming health policy will follow when evidence is presented in a well-packaged format.

There is, not surprisingly, a general acceptance among scholars and policy makers that research is not the sole source of influence on policy change. Policy makers have a wider context to consider and they have to 'invariably take politics, not just data into account' [41]. Vivian Lin in particular has defined three ‘competing rationalities’ which policy makers must balance to make decisions – technical, political, and cultural [14]. Policy processes are always political, and vested interests can have considerable effect over the scope to which it is possible even for sympathetic policy makers to prioritise issues or reform policy [8]. In the health sector, the interests of various stakeholders such as politicians, public servants, religious groups, pharmaceutical and diagnostic companies, and health professionals may often run counter to the introduction of new research findings, thus affecting policy making, budgeting, and implementation. Vested interests, combined with capacity constraints, can hamper evidence-based reform of health systems.

In terms of assessing research impact on policy, a recent multi-sectoral review undertaken by Boaz and colleagues found that most studies tend to adopt a case study approach and utilise qualitative methods [12]. They also noted how such studies can be either forward looking (tracking the contribution a specific research project makes to policy or practice) or backward looking (focusing on a specific policy change and investigating which research evidence was used, and how). However, the majority of impact assessment studies reveal that analysing attribution and impact of research is certainly not an easy task, largely due to the ‘uncertainty in determining a causal link between research and the outcome of a policy or the value of a policy outcome’ [42]. Most studies make little attempt to meaningfully deal with the issue of attribution beyond key stakeholders’ perceptions. The challenge here is while stakeholders may have unparalleled knowledge of poorly-documented processes, they are also likely to be biased. Triangulating data from different stakeholders, and with whatever published and unpublished documentary evidence is available, is therefore important for verifying stakeholder perceptions. Furthermore, the timing of impact assessment is an issue as there may be significant time lags to a policy change. As Garrett notes, 'the concrete results of policy research… may take time to emerge', suggesting that impact assessment takes place after some time lapse [43]. However, policy engagement in public health is often present at all stages of a research project, and it is thus conceivable for some kinds of research impacts to occur even while projects are ongoing.

### An analytical framework for factors shaping research impact on policy

Section 2 highlighted the complexity in defining research impact, as well as the diversity in the range of theories and approaches found in the literature to explain policy change. In this section we synthesise key elements from this literature that researchers can use to understand their role in policy processes, and introduce a framework to analyse the factors shaping the impact of research on policy. Across multiple studies and sectors – including efforts to synthesise what is known about evidence use in development [6,29], or works reviewing policy analysis theories in health [1,35], policy change is seen to be driven by the interaction of three dimensions, which can be combined to produce a synthesis approach:

1. *Policy ideas/narratives* – including how evidence and the health issue are conceptualised, and the way their relevance is understood with regard to policy agendas.

2. *Policy actors/networks* – including work emphasising the importance of actor interests, key decision makers and policy entrepreneurs, or networks and groups who are influential in decision making.

3*. Political context/institutions* – including the ‘hard’ structures in which decisions are made, as well as the broader ‘soft’ socio-economic, political and cultural environments which shape policy processes (e.g. the formal/informal ‘rules of the game’).

All three of these dimensions can interrelate, or can influence each other. For example, the policy context may influence which policy ideas are dominant and affect the relationship between researchers and policy actors. Research can affect or target each of the three dimensions, but there are a number of factors mediating its impact. Drawing on Jones and Sumner [44] and Sumner and colleagues [45], it is possible to separate these factors into two broad categories: first, those that are given or pre-established in a policy arena, i.e. the ‘pre-conditions’ for research impact on policy; and second, those related to researchers’ ‘interventions’ for research impact on policy.

The ‘pre-conditions’ for research impact on policy are factors that are given in a particular policy arena or context. These can include broad structural political contexts – such as the existence of responsive or transparent governance mechanisms [46] – or more specific elements of the political system, such as the existence of national development strategies, levels of decentralisation, or the roles played by civil society [47].

In contrast, researchers are also able to exert agency over particular actions and strategies – ‘interventions’ – in order to increase the probability of research impact in policy and practice throughout the research cycle. Possible actions might include packaging the research to have resonance with prevailing debates or increasing the researcher’s involvement in policy and practice communities and networks. Researchers can also work towards establishing a conducive environment in terms of organising regular meetings and consultations with key policy bodies. Table 1 summarises these pre-conditions for research impact on policy and how researchers and communications specialists can act or strategise within these to support research to policy engagement.

### Research impact in SRH policy and practice

The earlier sections of this paper highlighted a wide range of concepts and theories that can provide insights into research impact on policy. The field of SRH provides its own unique context in which those issues might play out. SRH represents a health field with particularly strong interest group involvement. Decisions in this field can also range from seemingly apolitical technical decisions around choice or timing of drug treatments, to dramatically politicised debates around what is considered sexually ‘normal’ or ‘acceptable’. In this section we discuss how the synthesis developed above may be applied to analysis of the factors affecting research uptake in this specific field. In Additional file 1 we utilise the analytical framework we presented in Table 1, illustrating how the framework can be applied to findings from many of the case studies in this supplement (Additional file 1). As explained in Section 3, the three dimensions of our framework (policy ideas, policy actors/networks and policy context) can interrelate in practice, and we draw out some of the examples of this in our discussion of Additional file 1.

#### Policy Ideas/Narratives

The first section of our framework is concerned with the pre-existing policy ideas, narratives and discourses relating to the specific SRH issue, and the actions that researchers can take to draw on or influence these ideas and discourses. Both SRH and HIV/AIDS are primarily considered health issues. As such, much of the discourse and policy debate is framed within a public health paradigm. Yet the nature of SRH and HIV/AIDS, however, means that decisions made within these areas can often have impacts on a range of other sectors and interest groups. In particular, these issues touch on aspects of sexuality, gender, and value judgements about behavioural norms and ‘morality’ held by policy actors and society at large. Some of the most obvious examples would be decisions around abortion services, promotion of different sexual behaviours (e.g. condom use) or non-sexual behaviours (i.e. abstinence) for HIV prevention, or engagement with individuals undertaking activities that are criminalised in many countries (such as injection drug use or commercial sex). Sexuality and reproduction are core issues for groups concerned with gender and human rights, and are often similarly important for organised religious and civil rights groups. As such, SRH policy issues may often serve as a point of convergence, or indeed potential confrontation, between actors and networks of actors from very different ideological and disciplinary backgrounds.

In their paper, ‘Social Construction of Target Populations’, Anne Schneider and Helen Ingram argue that the nature and social construction of target populations have important implications for policy outcomes, such as elements of the design or content [51]. By this they mean the attribution of shared characteristics of a discrete group, which have either positive or negative social connotations. The groups include powerful, positively constructed groups, labelled advantaged; powerful, negatively constructed groups, labelled contenders; weak but positively constructed groups, labelled dependents and finally weak and negatively constructed groups, labelled deviants. Users of SRH services in low income countries often face significant difficulties in organising themselves collectively to call for improvements in services (for a discussion of this in relation to users of contraceptives, see [52]), and tend to fall into either of the ‘weak’ categories. For example, pregnant women will often have limited influence on policy, but are likely to be portrayed in a positive or deserving light, while groups such as drug users, sex workers or men who have sex with men are often labelled as deviants [53], reducing the likelihood that policy makers will act on research showing the need for improved services for these groups. However, framing the issue in a different way or showing how it relates to existing national public health policy priorities may in some cases help to overcome political reluctance to target services to marginalised groups. For example, such a change occurred in Switzerland when provision of needles to injecting drug users was reframed from a policy of drug maintenance to a policy of harm reduction for HIV [54].

Additional file 1 shows some of the actions that researchers can take in order to address prevailing narratives and discourses when communicating their research. These include framing their research findings in ways that resonate with popularly held ideas in order to make the findings more attractive to policy makers. Appealing to prevailing discourses may be more effective in cases where the issues at stake are neglected but not too controversial [17,64]. They also include seeking to change ideas and discourses which research evidence shows to be harmful to health outcomes, for example by changing the way policy makers think about marginalised groups. Additional file 1 presents examples of taking a longer-term approach to encouraging public dialogue about sexuality in Bangladesh. Researchers made use of the fact that academia is commonly viewed by as having a high level of legitimacy in Bangladesh to create spaces for challenging dominant societal values [56].

The dominant public health paradigm that often underlies SRH discussions derives from the disciplines of medicine and epidemiology – both positivistic in outlook. Therefore, the need to frame arguments and policy recommendations as ‘evidence-based’ is typically present. This can often be seen when actors from non-public health backgrounds – such as advocacy groups, think tanks, religious groups, or lobbyists – use this language in making arguments. In another example in Additional file 1, The Pleasure Project, an advocacy and research organisation, carried out communications activities to raise awareness among policy makers about the evidence-base for the effectiveness of positive approaches to promoting safe sex with the aim of incorporating these approaches into mainstream HIV prevention [57].

#### Policy Actors/Networks

The second section of our framework focuses on the policy actors who are target audiences for research. The pre-existing characteristics of these target audiences include their receptivity to the research evidence, and the kinds of relationships and networks that researchers can use in communicating their research. The evidence generated by SRH research is varied, and different types of evidence may be perceived in different ways by particular target audiences and intermediaries. Those trained in health and other sciences typically adhere to a belief in a hierarchy of evidence. ‘Hard’ evidence is that which is seen as objective and quantitative. In contrast, ‘soft’ evidence is that which is subjective and qualitative [65]. Meta analyses of randomised controlled trials are at the top of the hierarchy (with the RCT commonly referred to as a ‘gold standard’ for evidence [66]), while anecdotal evidence is at the bottom [67].

Many of those groups concerned with the social, moral, and ideological issues touched upon by SRH may not make decisions or draw conclusions in the same way as the public health practitioners who attempt to follow a hierarchy of evidence (as defined by their own discipline), yet the institutional context in which many health decisions are made can often see that hierarchy in operation, if only in the background. There are often value judgements placed on evidence, or a need to frame evidence in particular ways, in order to satisfy the relevant decision makers. The context specificity, however, of many policies related to SRH – including nearly all interventions dealing with social factors, sexual behaviour or health systems – has, as mentioned, led to further concern regarding how to deal with so-called ‘complex interventions’ by public health practitioners [68]. The fact that such situations are typically seen as ‘special cases’ again reiterates the dominant positivist and causal paradigm that often exists in the health sector, as opposed to other social sciences which take such complexity and need for evidence beyond experimental trials as a starting point (e.g. [69]). The nature of the policy change and proposed intervention can thus have both political and complexity elements which need to be considered when understanding the roles and relative importance different types of evidence might play in influencing policy decisions.

The strength of networks and relationships that researchers can use to channel their research has a strong impact on research uptake. Crichton and Theobald show how pre-existing factors, such as the presence or absence of institutional links between the research institute and target policy actors, affect the ease of access to policy makers. In addition, researchers take action to create and strengthen relationships with policy actors, through establishing partnerships and building personal relationships [11]. For example, Additional file 1 provides examples of researchers and human rights lawyers collaborating to create alliances with sympathetic champions in government and civil society in order to influence policy on services for survivors of gender-based violence in Ghana (see also [58]). The table also indicates cases where researchers have used combinations of formal meetings and informal interactions to build close working relationships with STI policy makers in Ghana [21] and journalists in Kenya [60]. In some cases, researchers and communications experts can help to facilitate communication on SRH issues between policy makers and other groups in civil society. For example, The Pleasure Project used research communications activities to improve understanding between public-health actors and the sex industry [57].

#### Policy Context/Institutions

Finally, Additional file 1 demonstrates some of the contextual issues that are important in research uptake in the field of SRH. There is undoubtedly some SRH research which is highly technical in nature, and for which a policy change would have minimal moral implications – such as changing from one anti-retroviral drug treatment regimen to another, or an alteration in health worker responsibilities in the treatment of childbirth complications. Yet this does not mean these will be completely apolitical decisions. As discussed above, politics and vested interests have an important impact on health policy making and may act as disincentives for evidence-based policy. The nature of the particular SRH issue in question and the political, economic and social implications of policy change in that area have important influences on the responsiveness of policy audiences and the ‘room for manoeuvre’ policy makers have to use research evidence [8]. For example, the size of a potential policy’s impact, the types of people who would benefit or lose from its introduction, and the social construction of the issue and the target groups affected all affect research uptake. Some research issues draw in an enormous range of actors with competing interests and divergent attitudes to issues such as sexuality and gender. In such cases, policy makers will face a range of what Lin has described as ‘competing rationalities’ [14]. An example of this would be policies to provide adolescent girls with an HPV vaccine to prevent cervical cancer. While epidemiological evidence exists on the incidence of cervical cancer, the proportion linked to strains of HPV, and the efficacy of the vaccine, the ultimate political decision will equally be influenced by arguments around adolescent sexuality, women’s and girls’ rights, and the role of the state around those issues.

As aforementioned, contextual factors can interact with the other dimensions of the framework. For example, politics can influence the creation and propagation of particular ideas or narratives that may be harmful to health outcomes. In highly politically charged debates, there are cases where some stakeholders may spread incorrect information or even deliberate ‘misinformation’ about SRH issues. For example, on occasions when groups have exaggerated the dangers of medical abortion or overstated the side effects of contraceptives in order to achieve their political or social aims. One noteworthy example of this came in 2003 from a cardinal at the Vatican, who wrote a report stating how latex condoms may allow passage of particles larger than HIV viruses, and that HIV increases with the numbers of condoms distributed [70].

Additional file 1 gives some examples of the kinds of approaches SRH researchers can use to address contextual constraints to research uptake. In South Africa, researchers addressed the suspicion and lack of understanding of research in their study communities by using innovative consultation and communication techniques in order to facilitate the transfer of research-based knowledge to these communities (see also [63]). Researchers can also use opportunities arising within the policy process at specific points in time to influence policy. Researchers in Zambia used the opportunity to review draft national guidelines on ART therapy to recommend that their research findings on cotrimoxazole should be incorporated in Zambia’s national HIV treatment policy (see also [55]).

## Conclusions

This paper reviewed the literatures on international public health, policy analysis, and research impact evaluation to examine the uptake of evidence in the field of sexual and reproductive health. However, collaborative research projects involving public health researchers and political scientists working on international development or research impact evaluation remain relatively rare, despite the scope for greater cross-fertilisation of concepts, models and experience between them.

Review of existing work and analysis of the case studies in this supplement has highlighted how, when considering factors enabling research impact, it is helpful to distinguish between those that are ‘pre-existing’ (part of the policy context) and the actions and strategies (‘interventions’) deployed by researchers and communications specialists. While the latter group are less focused on more traditionally retrospective policy analysis, there are increasing examples of frameworks and approaches which take more prospective approaches, looking at what researchers can do to improve the uptake of research results (such as the Overseas Development Institute’s RAPID programme, and much work by Lavis and colleagues [5]). We argue that the second set of factors deserve increased attention from policy analysts, not least because of their usefulness for future efforts to influence policy with research. The analytical framework introduced in this paper can be used by researchers to examine the policy landscape affecting the uptake of research in specific settings, in order to guide attempts to ensure uptake of their findings. The case study papers in this supplement contain both examples of research on the use of evidence in policy making, as well as examples exploring the roles played by researchers and communications staff in influencing policy.

## Competing interests

The authors declare that they have no competing interests.

## Authors' contributions

AS, ST and JC conceived the initial design of the study. AS created the conceptual framework and wrote the first draft of sections of the paper. JP and JC wrote sections of the paper and revised drafts. ST and EZ revised drafts of the paper.

## Supplementary Material

Additional file 1 What determines policy outcomes and research use in SRH and HIV policy change? Click here for file
