# Supplementary material for: What shapes research impact on policy? Understanding research uptake in sexual and reproductive health policy processes in resource poor contexts
Source: Health Res Policy Syst. 2011 Jun 16;9(Suppl 1):S3. doi: 10.1186/1478-4505-9-S1-S3 (PMC3121134; doi:10.1186/1478-4505-9-S1-S3)
Supplement: Additional file 1 — What determines policy outcomes and research use in SRH and HIV policy change? [file 1478-4505-9-S1-S3-S1.PDF]

## Additional file 1. What determines policy outcomes and research use in SRH and HIV policy change?

| **What determines policy outcomes?** | **Determinants of research impact on SRH policy** | |
|---|---|---|
| | **Examples of ‘pre-conditions’ for research impact in SRH/HIV policy** | **Examples of ‘interventions’ to increase probability of research impact in SRH/HIV policy** |
| **Policy ideas, narratives and discourse(s)** | Awareness of research evidence for the efficacy of cotrimoxazole in Zambia, but lack of prioritisation on policy agenda [54].<br><br>Policy makers may be reluctant to acknowledge the existence of a problem because it is marginalised or stigmatised by prevailing societal norms and values. For example, although there are concepts about transgender identities in Bangladeshi culture, widespread discrimination and prejudice still exists [55]. | Researchers advocating for a change in perceptions to acknowledge cotrimoxazole as a policy issue [54].<br><br>Reframing SRH issues in accordance with prevailing norms can work in some cases where the issues at stake are neglected but not too controversial. In more controversial cases such as sexuality in Bangladesh, a more appropriate approach may be a longer-term strategy to raise awareness about the issue and challenge dominant societal values [55]. |
| | In many countries, influential national actors seek to delegitimize sexual and reproductive rights and frame them as ‘foreign’ and culturally inappropriate. External actors can lend support to issues that are controversial or neglected in national contexts by working in collaboration with local leaders or champions, eg sexuality in Bangladesh [55]. | Researchers in Bangladesh held attention-generating public events to create space for marginalised groups to voice their views and to encourage public dialogue on sexuality [55]. |

| | | |
|---|---|---|
| | Positive approaches to promote safe sex have not yet been incorporated into mainstream HIV prevention [56]. | Advocacy by The Pleasure Project on the evidence-base for the effectiveness of positive HIV prevention approaches and erotising safe sex education interventions [56]. |
| **Policy actors and networks** | In many policy contexts, there is a perception that policy should be evidence based, yet communication between policy makers and researchers may be hampered by a number of factors.<br><br>Some policy makers may act as 'champions' of particular policy issues. For example researchers employed in senior policy positions in government and influential NGOs pushed for a national policy on cotrimoxazole treatment in Zambia and Malawi [54]. | Researchers can act as champions for particular evidence-based policy change (Hutchinson et al). In other cases, they can create alliances with sympathetic and influential champions from within government and civil society (see [57].<br><br>Researchers and communications specialists can seek to build formal and informal networks with target audiences, often through establishing working relationships and friendships between individuals in both spheres. This facilitated the reform of STI policy in Ghana [20].<br>Researchers' credibility, historical integrity, relationships, communications and choice of terminology shaped the ways in which research was able to highlight the importance of HIV In Swaziland and determine the issue's take-up in policy circles [58]. |
| | Health services users often have limited influence in low and middle income countries, particularly when they are stigmatised in some way. Existence of a non-heterosexual subculture, but lack of public voice and lack of attention from policy makers [55].<br><br>Limited coverage of sexuality and SRH issues in the Kenyan media [59].<br><br>Lack of communication or understanding between public health actors and the sex industry [56]. | Networking between diverse stakeholders to create a policy community committed to sexuality and rights [55].<br><br>Use of formal meetings, informal interactions and two-way training to build understanding and trust between researchers and the media [59].<br><br>Communications activities by The Pleasure Project to raise understanding between public health actors and the sex industry in order to promote pleasure-focused approaches to safe sex [56]. |

| | | |
|---|---|---|
| | Capacity to use research evidence may vary between different sections and departments of government. Policy makers may be less willing to use research that they were not involved with from the start. | Close engagement with policy makers at all stages of research can help to overcome limited capacity or willingness to use research. |
| | In both lower and higher income countries, gaps in communication and understanding between researchers and key policy makers and practitioner audiences are common [56,60,61]. | 'Multidirectional information exchange' between researchers, practitioners and policy makers allows the sharing of knowledge between researchers, practitioners and policy-makers, improves the policy-relevance of research, and enables the identification of opportunities for research influence arising in policy processes [60,61]. |
| **Context and institutions** | External donors can impede or facilitate uptake of research by national policy makers [20]. This can lend support to issues that are controversial or neglected in national contexts, eg sexuality in Bangladesh [54]. | |
| | Community members and health services users may have limited voice or knowledge about research in many contexts [60,62]. | Researchers in Ghana overcame barriers to research uptake in study communities by building partnerships with community organisations and creating accessible communications pieces [60]. Creating community consultation committees and using innovative consultation and communication techniques helped to facilitate the transfer of research-based knowledge to participants in a randomised controlled trial in South Africa [62] |
| | The increasing politicisation of HIV policy and concern about high mortality rate among HIV positive TB patients created political pressure for policy makers to take action to ensure appropriate treatment in Zambia [54]. | Zambian researchers made use of the opportunity to review draft national guidelines on ART therapy to recommend the inclusion of cotrimoxazole in Zambia's national HIV treatment policy [54]. |
| | | |

Note: examples in this table are taken from other papers in this special issue [20,54-62].
